# Drug-Drug Interaction Extraction via Recurrent Hybrid Convolutional Neural Networks with an Improved Focal Loss

**DOI:** 10.3390/e21010037

**Published:** 2019-01-08

**Authors:** Xia Sun, Ke Dong, Long Ma, Richard Sutcliffe, Feijuan He, Sushing Chen, Jun Feng

**Affiliations:** 1 Department of Information Science and Technology, Northwest University, Xi’an 710127, China; 2 Department of Computer Science, Xi’an Jiaotong University City College, Xi’an 710069, China; 3 Department of Computer Information Science and Engineering, University of Florida, Gainesville, FL 32608, USA

**Keywords:** drug-drug interaction, convolutional neural network, dilated convolutions, cross-entropy, focal loss, relation extraction

## Abstract

Drug-drug interactions (DDIs) may bring huge health risks and dangerous effects to a patient’s body when taking two or more drugs at the same time or within a certain period of time. Therefore, the automatic extraction of unknown DDIs has great potential for the development of pharmaceutical agents and the safety of drug use. In this article, we propose a novel recurrent hybrid convolutional neural network (RHCNN) for DDI extraction from biomedical literature. In the embedding layer, the texts mentioning two entities are represented as a sequence of semantic embeddings and position embeddings. In particular, the complete semantic embedding is obtained by the information fusion between a word embedding and its contextual information which is learnt by recurrent structure. After that, the hybrid convolutional neural network is employed to learn the sentence-level features which consist of the local context features from consecutive words and the dependency features between separated words for DDI extraction. Lastly but most significantly, in order to make up for the defects of the traditional cross-entropy loss function when dealing with class imbalanced data, we apply an improved focal loss function to mitigate against this problem when using the DDIExtraction 2013 dataset. In our experiments, we achieve DDI automatic extraction with a micro F-score of 75.48% on the DDIExtraction 2013 dataset, outperforming the state-of-the-art approach by 2.49%.

## 1. Introduction

A drug-drug interaction (DDI) is a combined effect produced by taking two or more drugs at the same time or within a certain period of time. Adverse drug reactions (ADRs) may bring huge health risks and dangerous effects to a patient’s body [1]. Therefore, increasing our understanding of unknown interactions between drugs is of great importance in order to reduce public health safety accidents. With the increasing research efforts of medical practitioners on DDIs, a large amount of valuable information is buried in a body of unstructured biomedical literature which is growing exponentially. Currently, many DDIs can be found in publicly available drug-related databases such as Drugbank [2], PharmGKB [3], Drug Interaction database [4] and Stockley’s Drug Interactions [5]. However, the most common way to update databases is still to rely on human experts; this is time-consuming and dependent on the availability of suitable experts who are able to find DDIs from many sources and keep up-to-date with the latest discoveries. Therefore, the automatic extraction of structured DDIs from the overwhelming amount of unstructured biomedical literature has become an urgent problem for researchers.

DDI extraction is a typical relation-extraction task in natural language processing (NLP). Early DDI extraction was relatively rare due to the lack of annotated gold standard corpora. In recent years, with the success of the DDIExtraction 2011 [6] and DDIExtraction 2013 [7] challenges, a well-recognized benchmarking corpus was provided to evaluate the performance of various DDI extraction methods, which greatly stimulated the research enthusiasm of researchers and played an important role in the development of DDI tasks. The challenge of DDIExtraction 2013—*Extraction of drug-drug interactions from biomedical texts* is to classify the interaction types (*Mechanism*, *Effect*, *Advice*, *Int* and *Negative*) between two drug entities in a sentence from the biomedical literature. For instance, given the example sentence [8]

“[Pantoprazole]${}_{e1}$ has a much weaker effect on [clopidogrel]${}_{e2}$’s pharmacokinetics and on platelet reactivity during concomitant use.”

with annotated target drug entity mentions

*e*_1_ = “pantoprazole” and *e*_2_ = “clopidogrel”

the goal is to automatically recognize that this sentence expresses a *Mechanism* DDI type between $e_{1}$ and $e_{2}$.

In recent years, considerable efforts have been devoted to DDI extraction; existing methods can be divided into two categories: rule-based methods and machine-learning-based methods. The rule-based methods [9,10] use manually pre-defined rules which match expression patterns in the labelled text which correspond to DDI pairs. The limitation of these methods is that the performance will depend heavily on the ability of professionals and domain experts to devise large bodies of rules.

Compared with rule-based methods, machine-learning-based methods usually show better performance and generalization. These methods regard DDI extraction as a standard supervised learning problem. Various types of features are extracted and fed to a classifier, for example context features [11], shortest path features, domain knowledge features [12], heterogeneous features [13], a combination of word, parse tree and dependency graph features [14], integrated lexical, syntactic and phrase auxiliary features [15], and so on. The biggest challenges of feature-based machine learning methods are firstly to choose good features which result in effective learning, and secondly to extract those features accurately from a biomedical text.

With the improvement in computing power, the availability of large data sets and the continuous innovation of algorithms, deep learning technology has developed rapidly, and has achieved great success in many health data analysis tasks. The Convolutional Neural Network (CNN) is well-known deep learning approach. Morabito et al. [16] employed CNNs to detect early-stage Creutzfeldt-Jakob disease (CJD) from other forms of rapidly progressive dementia (RPD) and achieved outstanding experimental results. Acharya et al. [17] presented a novel computer model for EEG-based screening of depression using CNNs, which extended the diagnosis of different stages and levels of severity of depression. Yu et al. [18] proposed a CNN-based method to segment small organs from abdominal CT scans, hence enabling computers to assist human doctors for clinical purposes. Rajpurkar et al. [19] developed an algorithm which employs a 121-layer convolutional neural network to detect pneumonia from chest X-rays at a level exceeding practicing radiologists. Recently, some researchers have already successfully applied CNNs to DDI extraction and have shown better results than traditional statistical feature-based machine learning methods. CNNs aim to generalize the local and consecutive context of the sentence that mentions a DDI by using filters of different sizes. Liu et al. applied a CNN model to DDI extraction with word and position embedding [20]. Zhao et al. used a syntax word embedding to capture the syntactic information of a sentence and fed it into a CNN model for detecting DDIs [21]. Quan et al. used a multichannel CNN model with five types of word embedding to extract DDIs from unstructured biomedical literature [22]. Asada et al. encoded textual drug pairs with CNNs and encoded their molecular pairs with graph convolutional networks (GCNs) to predict DDIs [23], and obtained the best result so far for CNN-based DDI extraction methods. Although CNNs have been shown to perform well in the DDI task, due to the characteristics of a typical convolution operation, CNNs cannot capture the dependency between words or phrases which are some distance apart in a text. An example is a referring expression like “Azithromycin...” and an anaphoric reference to it later on in the text such as “it”, as shown in the following: “Azithromycin had no significant impact on the Cmax and AUC of zidovudine, although it significantly decreased the zidovudine tmax by 44% and increased the intracellular exposure to phosphorylated zidovudine by 110%.”

In addition to CNN models, Recurrent Neural Network (RNN) models have also been applied in DDI extraction. RNNs adaptively accumulate the contextual features in the whole sentence sequence via memory units [24]. Huang et al. presented a two-stage method, in which the first stage identified the positive instances using a feature-based binary classifier, and the second stage classified the positive instances into a specific category using a Long Short Term Memory (LSTM) based classifier [25]. Sahu and Anand used a joint RNN model with attention pooling to extract DDI information, and obtained a higher result than CNN-based methods [26]. Zhang et al. proposed a hierarchical RNN-based method to integrate the Shortest Dependency Paths (SDPs) and the sentence sequence for DDI extraction [27]. Zhou et al. employed the relative position information by means of an attention-based Bidirectional Long Short Term Memory (BiLSTM) to extract DDIs from biomedical texts [28]. This approach achieved an F-score of 72.99% which is the best performance so far on the DDIExtraction 2013 corpus. Although many methods have been proposed, DDI extraction research is still at an early stage and there is great potential for further improvements in its performance. However, four problems have first to be addressed:

Firstly, the embedding layers of existing models only use word embeddings to express the semantics of the text. However, polysemy is very common: in different contexts, the same word may have different meanings. Simply relying on word embeddings does not accurately express the actual meaning of the words. For example, the word “agent” shows different meanings in the following sentences; In “There is the risk of convulsions occurring in susceptible patients following the use of the new anaesthetic agents which are capable of inducing CNS excitability”, the word “agent” is referring to a drug. By contrast, in “All these years he’s been an agent for the East”, “agent” is referring to a person.

Secondly, most of the existing models that achieve better performance used off-the-shelf NLP toolkits to obtain stems, POS tags, syntactic chunk features [12], syntactic features [21], shortest dependency paths [27] and so on. Such tools may not be as accurate as they could be, because they are not tailored to the application domain; this can result in avoidable errors which propagate through the system and hence reduce its performance.

Thirdly, position embedding is the key information that reflects the core word information inside the sentence and allows the differences between the sentences in a DDI task to be distinguished [20,29]. Inappropriate setting of a model’s embedding vector dimension can cause effective information to be overwhelmed. For example, in the methods of Liu et al. [20] and Zhou et al. [28], the word embedding is set to 300 dimensions while position embedding is just 20 dimensions, meaning that important position information is almost ignored.

Lastly, the ratios of positive and negative instances in the original training set and test set (the DDIExtraction 2013 corpus) are 1:5.91 and 1:4.84 respectively. Obviously, the data set has a very serious class imbalance problem. Liu et al. [20] and Quan et al. [22] used negative instance filtering to alleviate class imbalance issues. The fundamental problem of class imbalance, however, remains. Therefore, other strategies are needed to solve this problem.

To address all four issues which we have described above, we propose a novel Recurrent Hybrid Convolutional Neural Network (RHCNN) for DDI extraction from biomedical literature. As stated in Lai et al. [30], a recurrent structure can capture contextual information. So we combine word embedding with the left- and right-context information of each word which is obtained by a BiLSTM model. Then we use the fully-connected layer for information fusion to obtain the final semantic embedding of the current word. Meanwhile, this operation will reduce the dimension of the semantic embedding, so that there is no large dimension gap between semantic information and position information; this avoids valuable position information being overwhelmed.

In addition, we propose a hybrid convolutional neural network that consists of typical convolutions and dilated convolutions to construct a sentence-level representation which contains both the local context features and the dependency features. The local and consecutive context features of the sentence that mentions DDI candidates are captured by typical convolution operations. The dependency features between separated words, such as are needed to link phrases or perform anaphora resolution, are extracted by dilated convolution operations. The architecture is based on the idea that dilated convolutions operate on a sliding window of context which need not be consecutive—the dilated window skips over every dilation of width d−1 inputs. Dilated convolutions have exhibited great potential for other NLP tasks such as machine translation [31], entity recognition [32] and text modeling [33]. To the best of our knowledge, it is the first time dilated CNNs have been used for DDI extraction.

The DDIExtraction 2013 corpus is divided into five categories, but the number of samples in each category is extremely imbalanced. Inspired by the work of focal loss in the image recognition field [34], we use the improved focal loss function for multiclass classification model training to avoid class imbalance and over-fitting. The proposed model (The experimental source-code is available at https://github.com/DongKeee/DDIExtraction) has been evaluated on the DDIExtraction 2013 corpus and achieves a micro F-score of 75.48%, outperforming the state-of-the-art approach [28] by 2.49%, thus demonstrating its effectiveness.

In sum, our key contributions are as follows:

- Our model does not rely on any NLP tools in the embedding layer, instead the sentences that mention DDI pairs are represented as sequences of semantic embeddings and position embeddings. In particular, we use the fully-connected layer for semantic information fusion between a word embedding and the contextual information that is learnt by recurrent structure; the two combined are employed to obtain a complete semantic representation of each word.
- We propose a hybrid convolutional neural network that consists of typical convolutions and dilated convolutions, and we believe it is the first time dilated CNNs have been used for DDI extraction.
- We introduce an improved focal loss function into the multiclass classification task in order to avoid class imbalance and the resulting over-fitting problem.
- We achieve state-of-the-art results for DDI extraction with a micro F-score of 75.48% on the DDIExtraction 2013 dataset, outperforming the methods relying on significantly richer domain knowledge.

## 2. Method

**Figure 1:**
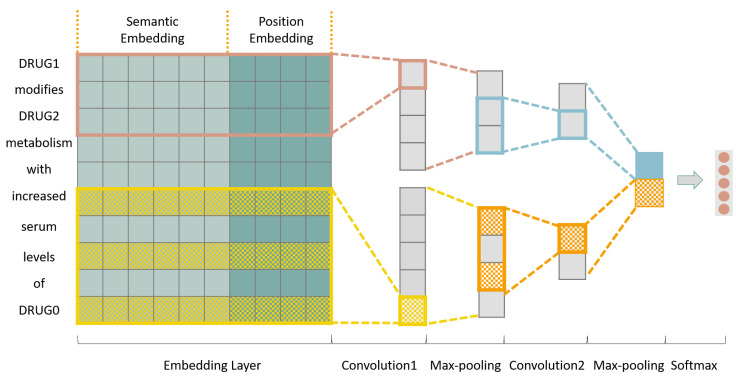
The architecture of recurrent hybrid convolutional neural network model. This figure is an example of the sentence after drug blinding “*DRUG1 modifies DRUG2 metabolism with increased serum levels of DRUG0.*” (DDIExtraction 2013 dataset, file Acetazolamide-ddi.xml, sentence DDI-DrugBank.d368.s0).

In this section, we present our recurrent hybrid convolutional neural network model. Figure 1 illustrates the overview of our architecture for DDI extraction. Given an input sentence *S* with a labeled pair of drug entity mentions $e_{1}$ and $e_{1}$, DDI extraction is the task of identifying the semantic relation holding between $e_{1}$ and $e_{1}$ among a set of candidate DDI types. In the word embedding layer, each word is represented by a semantic embedding and a position embedding. The semantic embedding is fused by the fully connected layer, which contains a word embedding for each word as well as the contextual features captured by the BiLSTM model from the word’s left and right context. These are combined to obtain a semantic vector for each word and hence narrow the dimension gap between semantic information and position information.

Next we use the hybrid convolutional neural network that consists of typical convolutions and dilated convolutions to construct the sentence-level representation which contains the local context features from consecutive words together with the dependency features between separated words. In the max pooling layer, the most important features are extracted from the output of the previous layer, and the dimensions of the features are reduced at the same time. Finally, we concatenate these features to form the sentence representation, and feed it to the fully-connected softmax layer for classification. Recall also that the final output is given by a new improved focal loss function. The remainder of this section will provide further details about this architecture.

### 2.1. Preprocessing

The DDI corpus contains two or more drug entities in each sentence. Given a sentence of *n* entities, there are $c_{n}^{2}$ DDI candidates which need to be classified. To allow generalization during learning and to keep only one drug pair remaining in each instance, we replace the two candidate entities with symbols “DRUG1” and “DRUG2” while all other drug entities are replaced by “DRUG0”, in common with previous methods [14,35]. Table 1 shows one example of a drug blinding. However, the result of the preprocessing operation is to generate a dataset with an imbalanced sample. For example, the sentence

“When *drugEntity1* or other hepatic enzyme inducers such as *drugEntity2* and *drugEntity3* have been taken concurrently with *drugEntity4*, lowered *drugEntity5* plasma levels have been reported.”

contains 5 drug entities, and three positive instances of DDI pairs: (*drugEntity1*, *drugEntity4*), (*drugEntity2*, *drugEntity4*) and (*drugEntity3*, *drugEntity4*). Thus the sentence reflects the existence of interactions between the stated drug entity pairs, and the DDI type for each pair is one of *Mechanism*, *Effect*, *Advice* and *Int*. On the other hand, the $c_{5}^{2}-3$ i.e., 7 DDI pairs such as (*drugEntity1*, *drugEntity2*), (*drugEntity1*, *drugEntity3*), (*drugEntity1*, *drugEntity5*) and so on are all negative instances, meaning that the sentence does not reflect the existence of these interactions. So the number of negative samples (7) is significantly more than that of the positive samples (3). In order to alleviate the problem of class imbalance and prevent the resulting performance degradation, we use a negative instance filtering strategy to filter out as many negative instances as possible, based on manually-formulated rules proposed by other researchers [14,20,22]. These rules can be summarised as follows:

- If a drug pair has the same name (like the instance shown in the third row of Table 1) or one drug is an abbreviation of the other entity, filter out the corresponding instances.
- If a drug pair are in a coordinate structure, filter out the corresponding instances.
- If one drug is a special case of the other, filter out the corresponding instances.

The statistics of the resulting dataset after negative instance filtering are shown in Table 2.

### 2.2. Embedding Layer

The input to our model is a sentence *S* that mentions two drug entities, where $S=\left\{w_{1},w_{2},\ldots ,w_{n}\right\}$, $w_{k}=$ “DRUG1” and $w_{t}=$ “DRUG2” ($k,t\in \left[1,n\right],k\neq t$). Each word $w_{i}$ of the sentence sequence is represented by both a semantic embedding and a position embedding.

Recently, word embedding has been successfully applied in various NLP tasks, such as sentiment analysis [36], relation classification [29], information retrieval [37] and so on. Word embedding refers to the mapping of words or phrases to real-value vectors, which must be learnt from significant amounts of unlabeled data. Various models [38,39,40] have been proposed to learn the word embedding. In order to obtain suitable high-quality vector space representations for biomedical NLP tasks, it is necessary to use a large number of articles in the biomedical field as training data. Unfortunately, it always takes too much time to train word embeddings. However, there are many pre-trained word embeddings that are freely available and of high quality [41,42]. So our experiments directly utilize the embedding provided by Pyysalo et al. [41], which is trained using articles from PubMed and the English Wikipedia.

**Figure 2:**
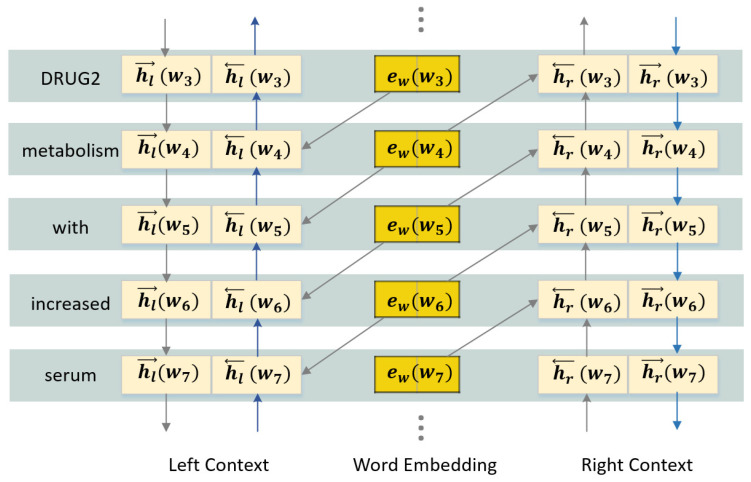
The bidirectional recurrent structure used for obtaining the actual semantic embedding of each word. This figure is a partial example of the sentence “*DRUG1 modifies DRUG2 metabolism with increased serum levels of DRUG0.*” (DDIExtraction 2013 dataset, file Acetazolamide-ddi.xml, sentence DDI-DrugBank.d368.s0).

Simply relying on word embeddings alone does not accurately express the actual meaning of a word due to the fact that it may have different meanings in different contexts, so we combine the embedding with contextual information to obtain fuller semantic information for each word. As depicted in Figure 2, the semantic embedding is generated by the bidirectional long short term memory model. Let $e_{w}\left(w_{i}\right)$, $e_{l}\left(w_{i}\right)$ and $e_{r}\left(w_{i}\right)$ denote the word embeddings, left-context embeddings and right-context embeddings. The left-side and right-side context embeddings are calculated by Equations (1)–(6):

(1) $$\vec{h_{l}}\left(w_{i}\right)=f\left({\overset{\to }{W}}_{l}^{in}e_{w}\left(w_{i-1}\right)+{\overset{\to }{W}}_{l}^{rec}\vec{h_{l}}\left(w_{i-1}\right)\right)$$

(2) $$\overset{\leftarrow }{h_{l}}\left(w_{i}\right)=f\left({\overset{\leftarrow }{W}}_{l}^{in}e_{w}\left(w_{i-1}\right)+{\overset{\leftarrow }{W}}_{l}^{rec}\overset{\leftarrow }{h_{l}}\left(w_{i+1}\right)\right)$$

(3) $$e_{l}\left(w_{i}\right)=\left[\vec{h_{l}}\left(w_{i}\right);\overset{\leftarrow }{h_{l}}\left(w_{i}\right)\right]$$

(4) $$\overset{\leftarrow }{h_{r}}\left(w_{i}\right)=f\left({\overset{\leftarrow }{W}}_{r}^{in}e_{w}\left(w_{i+1}\right)+{\overset{\leftarrow }{W}}_{r}^{rec}\overset{\leftarrow }{h_{r}}\left(w_{i-1}\right)\right)$$

(5) $$\vec{h_{r}}\left(w_{i}\right)=f\left({\overset{\to }{W}}_{r}^{in}e_{w}\left(w_{i+1}\right)+{\overset{\to }{W}}_{r}^{rec}\vec{h_{r}}\left(w_{i+1}\right)\right)$$

(6) $$e_{r}\left(w_{i}\right)=\left[\overset{\leftarrow }{h_{r}}\left(w_{i}\right);\vec{h_{r}}\left(w_{i}\right)\right]$$

where *f* is a non-linear activation function, $W^{\mathrm{in}}$ and $W^{rec}$ are weight matrices of the input and recurrent connections respectively. Then the semantic information can be represented as $S_{e}\left(w_{i}\right)=\left[e_{l}\left(w_{i}\right);e_{w}\left(w_{i}\right);e_{r}\left(w_{i}\right)\right]$, and the number of dimensions of it is $n_{s}=n_{l}+n_{w}+n_{r}$ where $n_{l},n_{w},n_{r}$ are the number of dimensions of the left-context embedding, word embedding and right-context embedding. To get the final semantic embedding $E_{s}\left(w_{i}\right)$ of each word, we fuse the word and contextual features by a fully connected layer, using Equation (7).

(7) $$E_{s}\left(w_{i}\right)=W_{s}S_{e}\left(w_{i}\right)+b_{s}$$

In order to capture significant information about the target entities, we combine semantic and position embedding. The position embedding is the key information that reflects the core word information inside the sentence and the differences between the sentences. In DDI extraction, given a sentence of *n* entities, there are $c_{n}^{2}$ DDI candidates which need to be classified, and these samples’ semantic embeddings are very similar. So it is necessary to specifically distinguish the target drug entities and magnify the differences between similar sentences. In our method, the position embedding $E_{pos}\left(w_{i}\right)$ is the combination of the vectors $e_{p1}\left(w_{i}\right)$ and $e_{p2}\left(w_{i}\right)$, which themselves are the relative distances of the word $w_{i}$ from the two marked entity mentions $w_{k}$ and $w_{t}$ [29,43] and which are then mapped to a randomly initialized vector of dimension $n_{d}$. So the final word embedding for $w_{i}$ is $E_{i}=\left[E_{s}\left(w_{i}\right);E_{pos}\left(w_{i}\right)\right]$ and is of size $N_{E}=n_{s}+n_{d}+n_{d}=n_{l}+n_{w}+n_{r}+2n_{d}$.

### 2.3. Hybrid Convolutional Layer

From the embedding layer we can obtain the local basic features for each word. In DDI extraction, the DDI types are judged by the semantic expression of whole sentences, so it is necessary to utilize all kinds of local features and to predict a candidate DDI type globally [29]. In our method, we use a hybrid convolutional neural network which consists of typical convolutions and dilated convolutions to obtain the sentence level representation.

The typical convolution operation is used to capture the local and consecutive contextual features of the sentence that mention DDI pairs, and the approach is expressed by Equation (8):

(8) $$Conv_{i}=ReLU\left(W_{f}E_{i:i+k-1}+B_{f}\right)$$

where *k* donates the filter size. $W_{f}\in R^{N_{E}\times k}$ is the filter applied to all possible consecutive context windows of *k* words. $B_{f\in }R$ is a bias term, and rectified linear units (ReLU) is a non-linear activation function. Then, a consecutive contextual feature vector $F_{conv}=\left[Conv_{1},Conv_{2},\ldots ,Conv_{n-k+1}\right]$ is obtained.

The dilated convolution operation is used to learn the dependency features between separated words in a text, such as a phrase and an anaphoric reference to it, and the method is expressed by Equation (9):

(9) $$D\_conv_{i}=ReLU\left(W_{D\_f}E_{i:i+\left(D\_k-1\right)\times d}+B_{D\_f}\right)$$

where D_k donates the filter size, *d* is the dilation size, $W_{D\_f}\in R^{N_{E}\times \left(D\_k-1\right)d+1}$ is the filter applied to all possible consecutive context windows of *k* words, and $B_{D\_f\in }R$ is a bias term. Then, an interval contextual feature vector from separated words $F_{D\_conv}=\left[D\_conv_{1},D\_conv_{2},\ldots ,D\_conv_{n-\left(k-1\right)\times d}\right]$ is obtained.

### 2.4. Max Pooling Layer

After the hybrid convolutional layer, the consecutive contextual features and the dependency features between the separated words are obtained. To determine the important local feature in each feature vector learnt by the prior layer and to reduce the computational complexity by decreasing the feature vector dimension, we use the max pooling operation to take the maximum value of each local feature of $F_{conv}$ and $F_{D\_conv}$, using Equations (10) and (11) where *w* is the window size of the pooling layer:

(10) $${\hat{F}}_{C\_i}=\max\left(Conv_{i:i+w-1}\right)$$

(11) $${\hat{F}}_{D\_i}=\max\left(D\_conv_{i:i+w-1}\right)$$

Then we will get the higher level features, denoted by $F_{c}=\left[{\hat{F}}_{C\_1},{\hat{F}}_{C\_2},\ldots ,{\hat{F}}_{C\_n-w+1}\right]$ and $F_{D}=\left[{\hat{F}}_{D\_1},{\hat{F}}_{D\_2},\ldots ,{\hat{F}}_{D\_n-w+1}\right]$. In our model, we use two layers of typical convolutions and dilated convolutions followed by a max pooling layer. When we get the final feature vectors $F_{c}$ and $F_{D}$ obtained by hybrid convolutions and a max pooling layer, the sentence vector $S=\left\{w_{1},w_{2},\ldots ,w_{n}\right\}$ will be represented as $s=[F_{c}$,$F_{D}]$.

### 2.5. Softmax Layer for Classification

In order to prevent the model from overfitting, we randomly drop out the neuron units from the network during the training process [44]. To do this, we randomly set some elements of *s* to 0 with a probability *p* and get a new sentence vector $S_{*}$. Then it will be fed to a fully-connected softmax layer in which the output size is the number of the DDI classes, and the conditional probability value *P* of each DDI type is obtained by Equation (12) where the softmax is a non-linear activation to achieve probability normalization:

(12) $$P\left(i|S,\theta \right)=softmax\left(W_{o}S_{*}+b_{o}\right)$$

### 2.6. Model Training

The following parameters of the RHCNN model need to be updated during training:

$$\theta =\left\{W^{in},W^{rec},W_{s,}b_{s,}e_{p1}\left(w_{i}\right),e_{p2}\left(w_{i}\right),W_{f,}B_{f,}W_{D\_f,}B_{D\_f,}W_{o,}b_{o}\right\}$$

Recently, the cross-entropy function is widely used as a loss function in the training of the existing DDI extraction models as shown in Equation (13):

(13) $$CE\left(p,y\right)=-\underset{i=1}{\sum^{C}}\;y_{i}\log\left(P\left(i\;\;\;|\;\;\;S,\theta \right)\right)$$

where *y* is the one-hot vector corresponding to the true category of the instance, and *C* is the number of DDI types. Cross-entropy reflects the gap between the predicted probability distribution and the true probability distribution. In model training, we expect the probability that the model predicts the sample to be as similar as possible to the true probability. As can be seen from the equation, if the predicted probability $P\left(i\;\;\;|\;\;\;S,\theta \right)$ that the model predicted its true category (when $y_{i}=1$) as close to its true probability distribution $y_{i}$ as we expect, the contribution of this sample to loss function (cross-entropy) will be reduced. Using cross-entropy as a loss function, aiming at minimizing the objective loss function will yield results that are consistent with our expectations. Therefore, cross-entropy is a commonly-used loss function in model training. In our experiments, the DDIExtraction 2013 corpus we used divides the samples into five categories (*Mechanism*, *Effect*, *Advice*, *Int* and *Negative*) and is imbalanced. The statistics of each category are shown in Table 2. As can be seen from the table, the *Negative* class has the largest number of samples. If we use cross-entropy as the objective function, it will give equal weight to all categories when calculating loss and cause huge interference to the learning process of the model parameters. A vast number of *Negative* class samples constitute a large proportion of the losses and hence dominate the direction of the gradient update so that the final trained model is more inclined to classify the sample into this type. However, it does not make much sense for us to divide the sample into *Negative* category to achieve a higher performance, because this category means that the text does not reflect the existence of interaction between two target drug entities. The DDI extraction task is more interested in knowing which interaction (*Advice*, *Effect*, *Mechanism* and *Int*) between drug entities is expressed in the biomedical literature.

Therefore, in order to solve the problems in the above cross-entropy function and avoid over-fitting, we use the improved focal loss function which consists of the focal loss together with the cross-entropy function for multiclass classification model training inspired by the work of focal loss in the image field [34] which has been used to solve the binary classification problem when data is imbalanced.

For notational convenience, we define $p_{t}$ as the corresponding $P\left(i\;\;\;|\;\;\;S,\theta \right)$ when $y_{i}$ is 1. So the cross-entropy loss function can be rewritten as Equation (14):

(14) $$CE\left(p,y\right)=CE\left(p_{t}\right)=-log\left(p_{t}\right)$$

The improved objective function is shown as Equation (15):

(15) $$L=-e\alpha {\left(1-p_{t}\right)}^{\gamma }\log\left(p_{t}\right)-\left(1-e\right)log\left(p_{t}\right)$$

where the left side of Equation (15) is the focal loss function proposed by Lin et al. [34], α is a weighting factor to balance the importance of samples from different classes ($\alpha \in \left[0,1\right]$), and is calculated by Equation (16), where $Count_{i}$ is the instance number of the *i*-th type. Under the action of α, we can flexibly calculate the weights of various samples according to the number of samples from different categories in the dataset, so that the final trained model has stronger ability to divide specific DDI categories than one using the traditional cross-entropy function. The α value of each category in the training set after the instance filtering of our experiment is shown in Table 3. As seen in table, the class with a small number of samples has larger weight and the type with a larger number of samples has smaller weight.

(16) $$\alpha_{i}=\frac{\frac{\sum_{i=1}^{5}Count_{i}}{Count_{i}}}{\sum_{i=1}^{5}\frac{\sum_{i=1}^{5}Count_{i}}{Count_{i}}}$$

The γ is a modulating factor for the improved focal loss function (γ>0), and smoothly adjusts the weight of easily-classified samples. For instance, when γ=2, a sample with the probability $p_{t}=0.9$ (such that ${1-p}_{t}=0.1$) has a loss contribution 100 times lower than one using a traditional cross-entropy function. Intuitively, the modulating factor reduces the loss contribution of examples that are easy to classify, so that more attention is paid to more difficult examples.

In addition, to prevent overfitting, we combine the focal loss with a traditional cross-entropy function [45], where *e* is a hyperparameter to adjust the weights of two functions. We optimize the parameters of the objective function L with the Adam Method [46] used in each training step. After the end of training, our model can classify the interaction types between two drug entities with an excellent performance.

## 3. Experiments

### 3.1. Datasets

We evaluated our RHCNN model on the DDIExtraction 2013 dataset, which is an established benchmark for DDI extraction [47,48]. The dataset is composed of 175 MEDLINE abstracts and 730 DrugBank documents, and has 5 distinguished DDI types, as follows:

1. Mechanism is used to annotate texts mentioning DDIs that describe the pharmacokinetic mechanism of two drug entities. e.g., *Probenecid competes with meropenem for active tubular secretion and thus inhibits the renal excretion of meropenem.*
2. Effect is used to annotate texts mentioning DDIs that describe an effect or a pharmacodynamic. e.g., *The action of Mecamylamine may be potentiated by anesthesia, other antihypertensive drugs and alcohol.*
3. Advice is used to annotate texts mentioning DDIs that give a recommendation or advice regarding a drug interaction. e.g., *Therefore, the coadministration of probenecid with meropenem is not recommended.*
4. Int is used to annotate texts mentioning DDIs that do not provide any additional information about when a DDI appears. e.g., *Ketoconazole: Potential interaction of Ketoconazole and Isoniazid may exist.*
5. Negative is used to annotate texts mentioning DDIs that do not reflect the existence of interaction between two target drug entities. e.g., *Drug-Drug Interactions: The pharmacokinetic and pharmacodynamic interactions between UROXATRAL and other alpha-blockers have not been determined.*

The corpus is divided into two parts: a training set and a test set, and the statistics of each set are shown in Table 2.

### 3.2. Experimental Setting

In our experiments, we use the Keras library with the backend of TensorFlow to implement our RHCNN model, and the code is written in Python3.6. Since the calculation of the CNN model requires a fixed data length and the actual length of each text instance is inconsistent, we set the maximum sentence length to 150. When the text length is less than this value, it is automatically padded with 0, otherwise the long sentence is cut to the maximum length. The dimensions of the word embeddings $n_{w}$, the left- and right-context embeddings $\;n_{l}$ and $n_{r}$, and the position embeddings $n_{d}$ are 200, 200, 200 and 15, respectively. The hyperparameters of our model are as follows. The maximal length of the sentence that mentions DDI pairs is set to 150. The hidden unit number of BiLSTM is set to 100. We use two kinds of filters for both typical and dilated convolution in which the filter sizes *k* are set to 3 and 5, and the dilation size *d* is set as 2. The factors *e* and γ in our objective function are set to 0.9 and 2. The batch size is set to 32, and the dropout rate is 0.5. The existing DDI extraction models are evaluated by micro-averaged F-scores. This metric is defined as Equations (17)–(19):

(17) $$\text{micro-P}=\frac{\bar{TP}}{\bar{TP}+\bar{FP}}$$

(18) $$\text{micro-R}=\frac{\bar{TP}}{\bar{TP}+\bar{FN}}$$

(19) $$\text{micro-F}=\frac{2\times \text{micro-P}\times \text{micro-R}}{\text{micro-P}+\text{micro-R}}$$

where $\bar{TP},\bar{FP},\bar{FN}$ denote the average value calculated across different classes of true positives, false positives and false negatives, respectively. In order to compare the RHCNN performance with other models, we also use the micro-averaged F-score to evaluate our models.

### 3.3. Performance Comparison with Other Methods

In this section, we compare the performance of our RHCNN model with other established methods. Table 4 shows the experimental results of different models on the DDIExtraction 2013 dataset. As the table shows, we calculate the overall Precision (P), Recall (R) and F-score to assess the performance of our model, and achieve scores of 77.30%, 73.75% and 75.48%, which are all higher than the current state-of-the-art method of Zhou et al. [28] by 1.5%, 3.37% and 2.49%, respectively. It can be seen that the performance of the neural network-based methods is generally higher than that of the traditional feature-based methods. This is because the former methods can learn efficient and useful features automatically, avoiding the time-consuming and laborious task of obtaining such features manually.

To assess the level of difficulty of detecting each type of interaction, we evaluate each DDI type separately. The F-score of the *Advice*, *Effect*, *Mechanism*, and *Int* types is 80.54%, 73.49%, 78.25% and 58.90% respectively. Among the four types, our RHCNN model performs best on *Advice* instances and worst on *Int* instances, the difference between the F-score on these two types being 21.64%. By comparison with other excellent models, our model achieves the highest performance yet on *Effect*, *Mechanism* and *Int* types, these being 1.49%, 1.93% and 4% higher than the highest records respectively.

After comparing and analyzing existing state-of-the-art models, our model achieves better performance mainly due to the following aspects. Firstly, instead of using features that need to be obtained by NLP tools, we use features that can be automatically obtained during the training process. In existing models with better performance, Zhao et al. [21], Huang et al. [25] and Zhang et al. [27] used the Enju parser [51], GDep [52] and the Stanford parser [53] respectively to parse the sentences and extract the important features, each achieving F-scores of 68.6%, 69% and 72.9%. However, these tools may not be entirely accurate, potentially leading to the propagation of errors which hinder the performance of these models.

Secondly, we combine contextual information with lexical features to obtain a more complete semantic representation of a word than has been used in this task hitherto. In the existing methods that only use the word embedding and position embedding as features [20,22,28], the semantic information in the feature only contains the word embedding. However, polysemy is a universal phenomenon of language: the meaning of a word may depend on its context of use. Therefore, using only word embedding information does not guarantee that the semantics are correctly expressed. We fuse the information of word and context to get a more complete semantic representation which can achieve an F-score 2.49% higher than best existing method.

Thirdly, the importance of position features has been enhanced by our method. The position features provide core information that can identify keywords within a sentence and expand differences between sentences. The dimensional differences between semantic embedding and position embedding are very large in the work of Liu et al. [20] and Sahu and Anand [26],which will cause the important position information to be ignored, resulting in a large impact on performance. Our method outperforms the best model of them (Sahu and Anand’s work) by 4% in micro F-score.

Fourthly and finally, we address the data imbalance problem in the DDI data set. As seen from Table 2, the existing advanced models generally have poor classification performance on the *Int* instances, mainly due to the data of the *Int* type being very small in the original DDIExtraction 2013 dataset. When evaluating each DDI type separately, our model has an F-score 4% higher than other approaches applied to the *Int* type, which is the highest degree of improvement compared to other categories. This is because we mitigate against the data imbalance problem by means of negative instance filtering and an improved loss function.

### 3.4. The Effect of the Strategies and Features on Performance

To further investigate the effectiveness of our strategies, we separate the effect of each strategy on the overall F-score, as shown in Table 5. When we remove the negative instance filtering strategy, the overall F-score of our model drops by 1.16%. The ratio of positive and negative instances in the original training set and test set is 1:5.91 and 1:4.84. As we have previously discussed, the original data set has a very serious data imbalance problem, which will make the classification result of the model shift to the category with more instances. After the negative instance filtering strategy is applied to filter out some distinct negative instances, the problem of data imbalance can be alleviated to some extent and the ratio changes from 1:5.91 to 1:4.76 and 1:4.84 to 1:3.83, respectively.

Next, we discover that the information fusion strategy is indispensable to the DDI classification problem as the F-score decreases by 1.09% when it is removed. After we use the BiLSTM model to obtain the left-side and right-side context information separately, if we simply concatenate them with the current word as $\left[e_{w}\left(w_{i}\right);e_{l}\left(w_{i}\right);e_{r}\left(w_{i}\right)\right]$, we will in fact get three independent features, which cannot fully capture the semantics of the current word. Then we use the fully-connected layer to fuse context and word information so as to get a more complete semantic representation of the current word.

After that, we change our improved focal loss function to the original cross entropy loss function and find that the F-score is reduced by 2.19%. The traditional cross-entropy function gives equal weight to all categories of instances. In the DDI dataset with its imbalance problem, the model is biased towards the category with the largest number of samples (i.e., the negative class), which seriously impairs performance. The improved focal loss used in our model obtains better performance, which addresses the data imbalance problem and over-fitting problem by a weighting factor α to balance the importance of different class samples, together with a modulating factor γ to reduce the loss contribution of examples that are easy to classify, so that more attention is paid to more difficult examples.

In order to verify the necessity of each feature, we compare the original model with the model that continuously adds new features. The experimental results are shown in Table 6. When only using the word embedding, our model achieves an F-score of 69.57%. When the position embedding, initialized randomly and learnt automatically, is integrated with the word embedding, the performance is further improved; this demonstrates the importance of position features in DDI tasks for identifying drug entities in similar sentences, highlighting the key information within sentences as well as the differences between sentences. Next, we use an LSTM and BiLSTM model respectively to obtain the contextual information. Because a BiLSTM learns both forward and backward information, it can obtain more complete contextual information, contributing to an F-score improvement of 5.28%.

Although our RHCNN model outperforms all other state-of-the-art methods on the DDIExtraction 2013 dataset, there is still some room for further improvements. As seen from Table 7 (where the numbers on the left and right sides of the plus signs represent the results of the RHCNN prediction and the results of the negative instance filter rules, respectively [20]), a total of 399 samples in our model are misclassified. Furthermore, 187 out of 979 positive instances are misclassified into negative instances, accounting for about 47% of the misclassified instances. In addition, 142 out of 4737 negative instances are misclassified into positive instances, accounting for about 36% of the misclassified instances. Seventy out of 979 instances are misclassified between four different categories of positive instances, accounting for about 17% of the misclassified instances. It can be seen from the above statistics that the main error lies in the misclassification between positive and negative instances. In addition, among the misclassifications between different categories of positive instances, 36 out of 70 instances (i.e., nearly 50%) occur when an *Int* type is wrongly classified as an *Effect* type, mainly because the number of *Int* type samples is small and the semantics between the two types are similar. Therefore, reducing the misclassification between positive and negative on the one hand, and misclassification between different positive instances on the other hand, will be the key issues that we need to solve in the future.

## 4. Conclusions

In this paper, we have proposed a novel method using a Recurrent Hybrid Convolutional Neural Network (RHCNN) for DDI extraction from biomedical literature. In our network, the contextual information is captured by recurrent structure, which is fused with word embeddings, resulting in a more complete semantic representation for each of the words. These representations are then sent to the concatenated word-level representation which consists of the semantic and position embeddings. These in turn form the input to a hybrid convolutional neural network so as to obtain the sentence-level representation which contains the local contextual features from consecutive words and the dependency features between interval words for DDI extraction. To the best of our knowledge, it is the first research to use dilated convolutions for the DDI extraction task. Last but not least, in order to make up for the defects of the traditional cross-entropy loss function when dealing with class imbalanced data, we apply an improved focal loss function to mitigate this problem. Our approach achieves an F-score of 75.48% on the DDIExtraction 2013 dataset, which outperforms the state-of-the-art approach by 2.49%.

## Figures and Tables

**Table 1:** An example for drug blinding preprocessing of sentence “*DIAMOX modifies phenytoin metabolism with increased serum levels of phenytoin.*” (DDIExtraction 2013 dataset, file Acetazolamide-ddi.xml, sentence DDI-DrugBank.d368.s0).

Drug Pair ($e_{1}$,$e_{2}$)	DDI Candidate after Drug Blinding
(DIAMOX, phenytoin)	DRUG1 modifies DRUG2 metabolism with increased serum levels of DRUG0.
(DIAMOX, phenytoin)	DRUG1 modifies DRUG0 metabolism with increased serum levels of DRUG2.
(phenytoin, phenytoin)	DRUG0 modifies DRUG1 metabolism with increased serum levels of DRUG2.

**Table 2:** The statistic of DDIExtraction 2013 dataset.

Types	Training Set		Test Set	
	Original	Filtered	Original	Filtered
DDI pairs	27,792	23,010	5716	4721
Positive	4020	3998	979	976
Negative	23,772	19,012	4737	3745
Advice	826	822	221	221
Effect	1687	1669	360	360
Mechanism	1319	1319	302	299
Int	188	188	96	96

**Table 3:** The α value of each category in the training set after the instance filtering.

DDI Type	The Number of Sample	α
Advice	822	0.15
Effect	1669	0.08
Mechanism	1319	0.10
Int	188	0.67
Negative	19,012	0.01

**Table 4:** Performance comparison with other state-of-art methods.

	Models	F-Score of Each DDI Type				Overall		
		Advice	Effect	Mechanism	Int	Precision	Recall	F-Score
	UTurku [12]	63.0	60.0	58.2	50.7	73.20	49.90	59.40
Feature-based	FBK irst [49]	69.20	62.80	67.90	54.70	64.60	65.60	65.10
method	Kim [14]	72.50	66.20	69.30	48.30	-	-	67.00
	Raihani [15]	77.40	69.60	73.60	52.40	73.70	68.70	71.10
	CNN [20]	77.72	69.32	70.23	46.37	75.70	64.66	69.75
	SCNN [21]	-	-	-	-	72.50	65.10	68.60
	MCCNN [22]	78.00	68.20	72.20	51.00	75.99	65.25	70.21
Neural	GRU [50]	-	-	-	-	73.67	70.79	72.20
network-based	CNN-GCNs [23]	81.62	71.03	73.83	45.83	73.31	71.81	72.55
method	SVM-LSTM [25]	71.50	72.00	73.80	54.90	75.30	63.70	69.00
	Joint-LSTMs [26]	79.41	67.57	76.32	43.07	73.41	69.66	71.48
	Hierarchical RNNs [27]	80.30	71.80	74.00	54.30	74.10	71.80	72.90
	PM-BLSTM [28]	81.60	71.28	74.42	48.57	75.80	70.38	72.99
Our method	RHCNN	80.54	**73.49**	**78.25**	**58.90**	**77.30**	**73.75**	**75.48**

**Table 5:** The effect of different strategies on performance.

Strategy	F-Score	Δ
basic RHCNN + improved focal loss function + information fusion + negative instance filtering	75.48	-
basic RHCNN + improved focal loss function + information fusion	74.32	−1.16
basic RHCNN + improved focal loss function + negative instance filtering	74.39	−1.09
basic RHCNN + cross-entropy loss function + negative instance filtering + information fusion	73.29	−2.19

**Table 6:** The effect of different features on performance.

Embedding Feature		F-Score	Δ
word		69.57	−
word + position		70.20	+0.63
word + context + position	context trained by LSTM	73.66	+3.46
	context trained by BiLSTM	75.48	+5.28

**Table 7:** Prediction results of our Recurrent Hybrid Convolutional Neural Network (RHCNN) method.

		Prediction Results					
	*Types*	*Advice*	*Effect*	*Mechanism*	*Int*	*Negative*	*Total*
True label	*Advice*	178	5	3	2	33	221
	*Effect*	2	269	9	0	80	360
	*Mechanism*	7	4	232	0	56 + 3	302
	*Int*	0	36	2	43	15	96
	*Negative*	34	58	45	5	3603 + 992	4737
	*Total*	221	372	291	50	4782	5716
